# β-Lactam-Induced Fever Mimicking Post-caesarean Infection: A Diagnostic Dilemma

**DOI:** 10.7759/cureus.105556

**Published:** 2026-03-20

**Authors:** Victor O Olagundoye, Obe John Ame, Najat Khenyab

**Affiliations:** 1 Obstetrics and Gynecology, Women's Wellness and Research Center, Doha, QAT; 2 Obstetrics and Gynecology, Women’s Wellness and Research Center, Doha, QAT

**Keywords:** caesarean section, drug fever, drug induced fever, postoperative fever, puerperal infection, β-lactam antibiotics

## Abstract

Fever after a caesarean section is often believed to be caused by infections, which usually require an escalation of antimicrobial treatment. Although caesarean delivery can be lifesaving for both mother and baby, the procedure carries risks, with emergency caesarean sections being associated with a higher chance of complications, including infection and haemorrhage, compared to elective caesarean sections.

Drug-induced fever is an important yet often overlooked alternative diagnosis in obstetrics and can be difficult to recognise in patients receiving antibiotics for suspected postoperative infection. Persistent fever in a postoperative patient is a significant concern, as it may indicate ongoing or worsening infection and require systematic evaluation and a multidisciplinary approach.

We report a case of a 31-year-old woman in her first pregnancy who developed a fever of 39.8°C, abdominal pain, and cough on day two following an emergency caesarean section. Her blood culture, urine culture, high vaginal swab, and viral panel were negative. Her white cell count (WBC), lactic acid, and procalcitonin levels were within normal limits, but C-reactive protein (CRP), a non-specific inflammatory marker, was the only elevated inflammatory marker. Pelvic ultrasound showed a small hematoma at the surgical site, and a chest X-ray indicated bilateral bronchopneumonia. A diagnosis of bronchopneumonia and pelvic haematoma was made. She was started on intravenous β-lactam antibiotics. Her cough resolved within two days, and she remained relatively well, but her high-grade fever persisted.

Despite escalating her antibiotics, she continued to experience a high-grade fever, which led to her transfer to a tertiary hospital. There, she was managed by a multidisciplinary team (MDT) of infectious disease specialists (ID team), pharmacists, physicians, and obstetricians, but her high-grade fever persisted. Repeat blood cultures, urine cultures, and high vaginal swabs were all negative. Similarly, her WBC, lactic acid, and procalcitonin levels, markers of infection, were within normal limits, but her CRP remained elevated. A further CT scan to exclude infected pelvic vein thrombosis or retained products of conception was negative, but raised concerns about worsening abdominal infection and possible peritonitis, even though the patient felt well with no abdominal pain or tenderness on examination. The CT report created diagnostic and management dilemmas. The MDT discussed the report extensively and recommended an exploratory laparotomy, which was explained to the patient and her family, but she declined. The next day, she developed jaundice and drug-induced hepatitis, leading to suspicion of drug-induced fever. All her antibiotics were stopped, and she became fever-free within days. The worsening ultrasound and CT scan findings are most likely due to an unusual response to a systemic hypersensitivity immune complex reaction to drug-induced fever rather than infection.

This case underscores the challenges of managing patients with persistent fever and highlights the importance of vigilance and awareness of drug-induced fever as a potential cause. Regular comprehensive assessments, including clinical examinations and investigations, are essential to identify infectious causes. A delayed diagnosis can lead to longer hospital stays, unnecessary tests, increased healthcare costs, and possible harm to the patient.

## Introduction

Postoperative fever is not uncommon in obstetric care. It is defined as a temperature above 38°C on two consecutive postoperative days or above 39°C on any postoperative day [1]. Identifying the cause of the fever can be challenging. Nosocomial infections, pelvic infections, and surgical site infections (SSIs) are the most common causes of postoperative infection. The global estimate of SSI following caesarean section is 7.0%, while it is 9-11% in the UK [2,3]. A systematic, multidisciplinary approach is crucial for achieving good clinical outcomes in patients with persistent fever.

Antibiotic-induced fever is rare in obstetrics, and it is often overlooked in the initial differential diagnosis of persistent postoperative fever. There is no consensus on the definition of drug fever. Luan et al. [4] defined drug-induced fever as an adverse febrile reaction that begins soon after starting a single drug and disappears after stopping the drug, once other causes have been excluded. Semeco et al. [5] outlined the criteria for drug fever as a febrile response that meets these conditions: 1) onset after drug administration; 2) resolution within 72 hours of stopping the drug without specific treatment; 3) no other cause identified through history, physical exam, laboratory tests, or imaging; and 4) no fever relapse within 72 hours after defervescence.

Antibiotics, anticonvulsants, antineoplastic agents, and cardiac drugs are the medications most commonly associated with drug-induced fever [6]. According to Johnson and Curha [7], the mechanisms behind drug fever include: 1) altered thermoregulatory mechanisms resulting from disruption of the body’s ability to regulate temperature; 2) febrile response triggered by the process of drug administration, such as vaccine administration, inducing febrile reactions; 3) fever related to the pharmacological action of a drug, such as Jarisch-Herxheimer reactions; 4) idiosyncratic reactions; and 5) hypersensitivity or immunological response, which is the most common mechanism of drug-induced fever. β-lactam antibiotics are the most frequently associated antibiotics with drug-induced fever. Many drugs in the β-lactam class can cause drug-induced fever because the mechanism usually involves immune-mediated hypersensitivity responses, which are not unique to a single agent as they share the same lactam ring structure.

This report describes a case of β-lactam-induced fever diagnosed after 10 days of antibiotic therapy, prolonged intensive care in a high-dependency unit (HDU), multidisciplinary team (MDT) management, thorough assessment, and multiple investigations, including blood and urine cultures and various imaging studies, for suspected pelvic infection following an emergency caesarean section during the second stage of labour. 

## Case presentation

A 31-year-old woman in her first pregnancy was transferred from a local hospital to a tertiary hospital on postoperative day five following an emergency caesarean section due to persistent fever and worsening radiological findings. Her antenatal care was uneventful. Her labour started spontaneously and progressed smoothly to full cervical dilation, but she underwent an emergency caesarean section under spinal anaesthesia for suspected fetal distress in the second stage of labour. The estimated blood loss was 350 ml. On postoperative day two, she reported a fever and a non-productive cough. On examination, her temperature was 39.8°C, blood pressure 128/76 mmHg, pulse rate 113 per minute, and respiratory rate 19 per minute. The caesarean section wound was clean, and her chest was clear. Blood tests showed a haemoglobin (Hb) of 8.6 g/dL (reference range 12-15 g/dL). Her preoperative Hb was 10.4 g/dL. We attributed the drop in haemoglobin to intraoperative blood loss. We did not consider this drop in haemoglobin to be significant enough to require a blood transfusion, nor did it contribute to her condition. We did not transfuse her because she was not symptomatic of anaemia. Her white blood cell count (WBC) was 10.5 x10^3/uL (reference range 4-10 x10^3/uL) and C-reactive protein (CRP) was 216 mg/L (reference range: 0-5 mg/L), lactic acid was 1.1 mmol/L (reference range: 0.5-2.2 mmol/L), and procalcitonin was 0.10 ng/ml (reference range: <0.5 ng/ml). The laboratory parameters from day two post-caesarean delivery through follow-up are shown in Table 1.

**Table 1:** Key laboratory parameters of the patient from pre-op up to follow-up. ALT: alanine aminotransferase, AST: aspartate aminotransferase, C/S: caesarean section, F/U: follow-up

Parameters	Normal Range	Pre-Op	Day 2 Post c/s	Day 5 Post c/s	Day 10 Post c/s	Day 16 Post c/s	At F/U
Haemoglobin (gm/dl)	12-15	10.4	8.6	7.4	8.0	9.1	10.7
White cell count (x10^3/ul)	4-10	8.7	10.5	6.7	12.4	9.3	5.8
C-Reactive Protein (mg/L)	0-5		216	302	191	93	2.1
Lactic acid (mmol/L)	0.5-2.2		1.1	1.1	1.1	1.1	1.0
Procalcitonin(ng/ml)	<0.5		0.1	0.1	0.16	0.06	0.06
ALT (u/L)	<32		23	25	234	68	23
AST (u/L)	<32		21	26	237	35	20
Bilirubin (µmol/L)	<21		12	14	59	40	11

**Figure 1:**
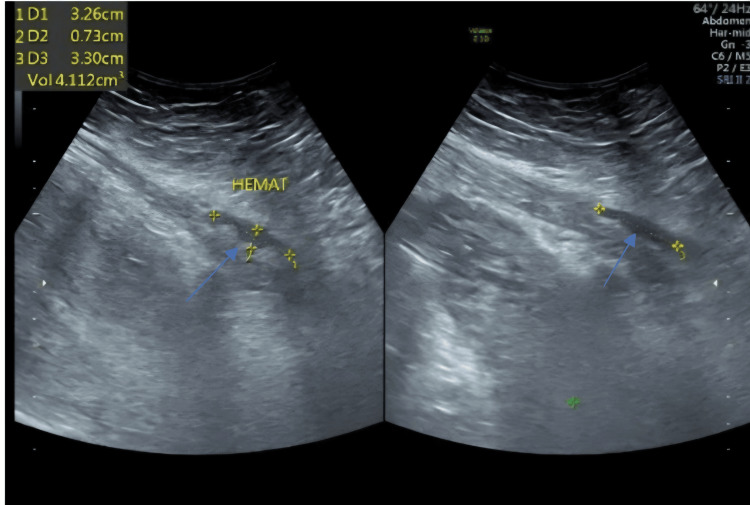
Pelvic ultrasound showing hematoma measuring 33x32x0.73 mm at the surgical site

**Figure 2:**
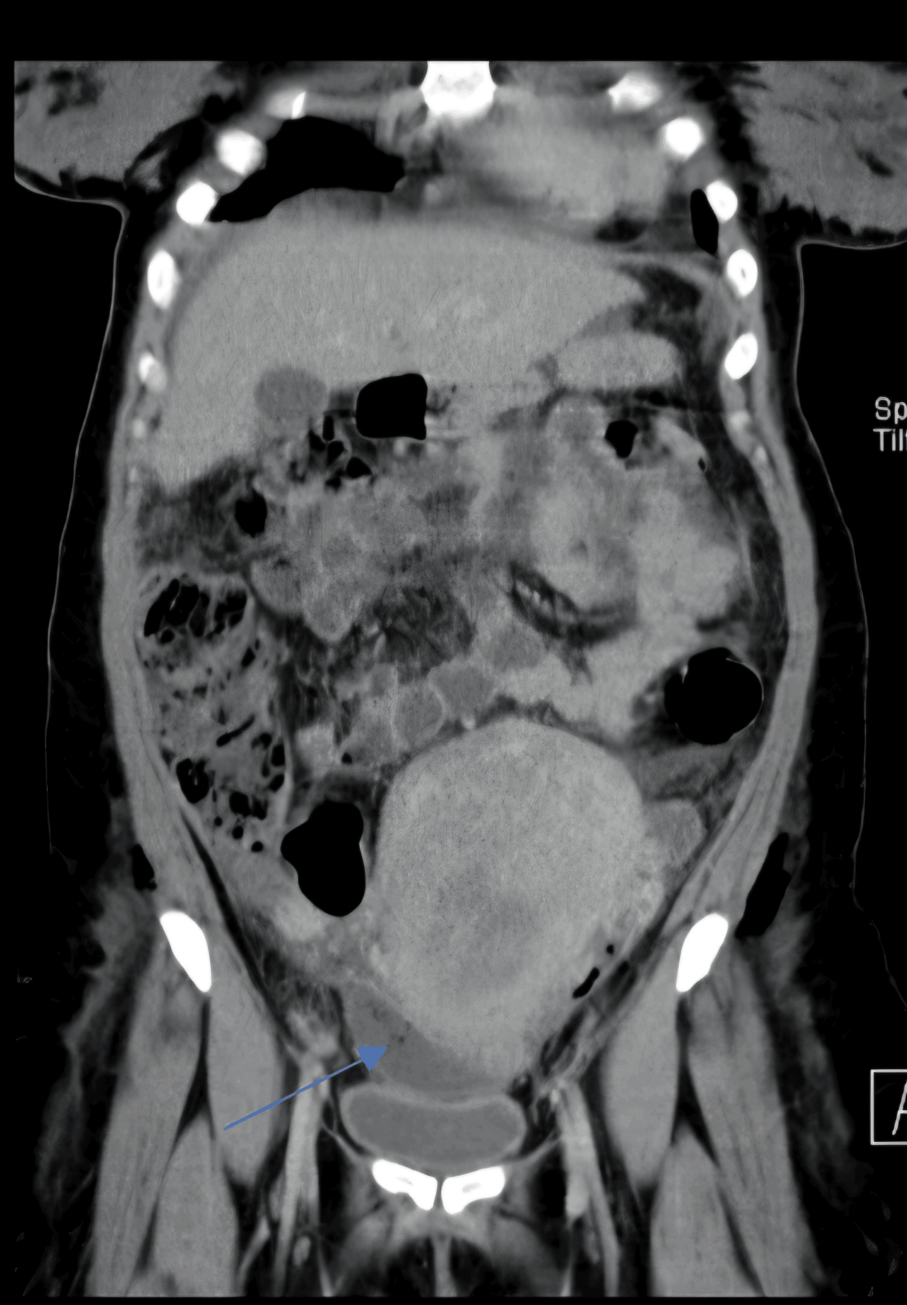
Computed tomography (CT) pelvis and abdomen showing hematoma at surgical site

**Figure 3:**
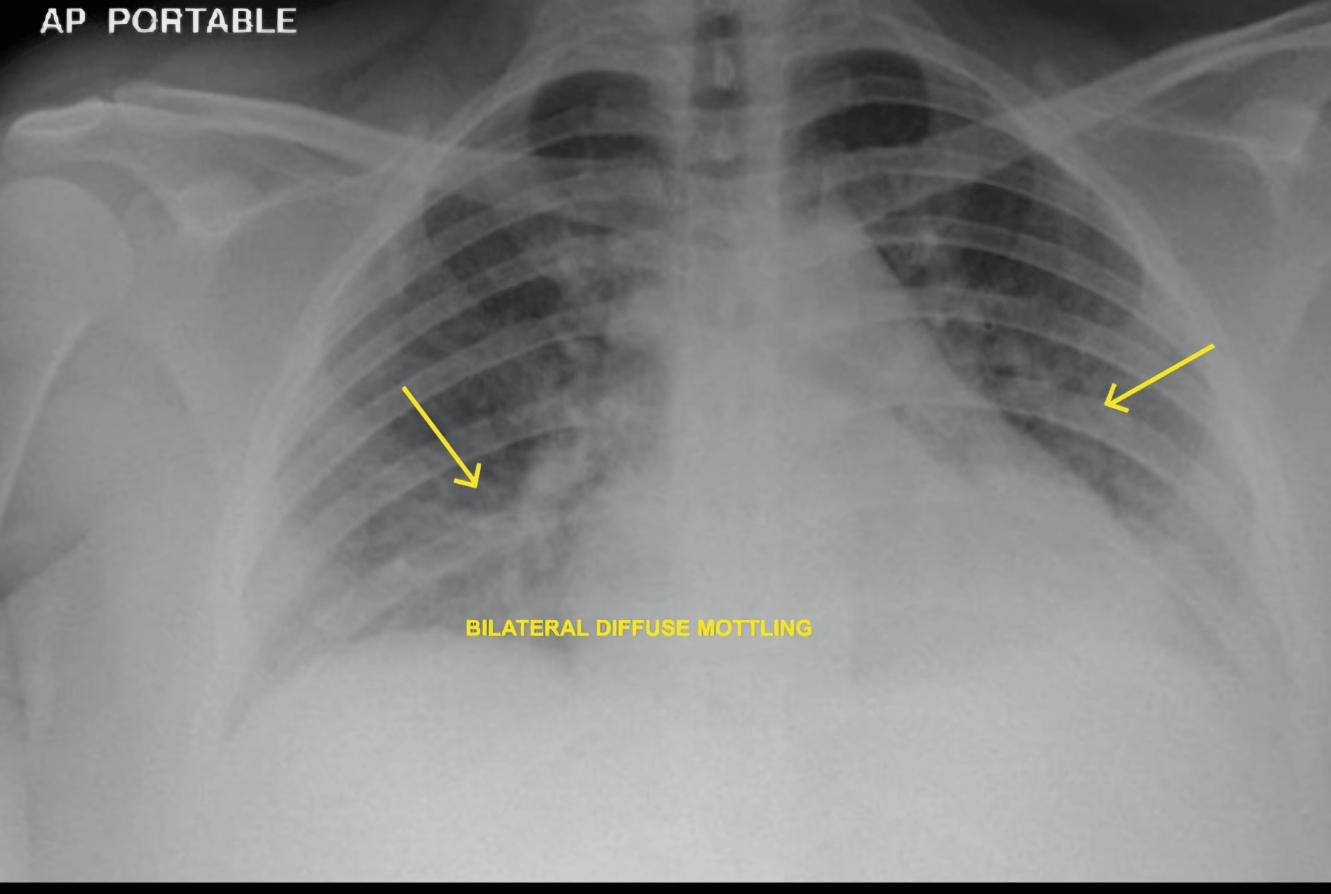
Chest X-ray showing bilateral diffuse mottling suggestive of bronchopneumonia

Pelvic ultrasound showed a small hematoma measuring 33x32x7.3 mm at the surgical site (Figure 1). This was confirmed with CT scan (Figure 2). Chest X-ray showed bilateral diffuse mottling suggestive of bilateral bronchopneumonia as shown in Figure 3. 

Blood culture, urine culture, and viral panel were negative. Our initial diagnosis was bronchopneumonia based on her symptom of cough and the chest X-ray report, along with a possible pelvic infection due to an increased risk of pelvic infection associated with emergency caesarean section and the finding of a pelvic haematoma on ultrasound and CT report. She was started on ceftriaxone and metronidazole.

**Figure 4:**
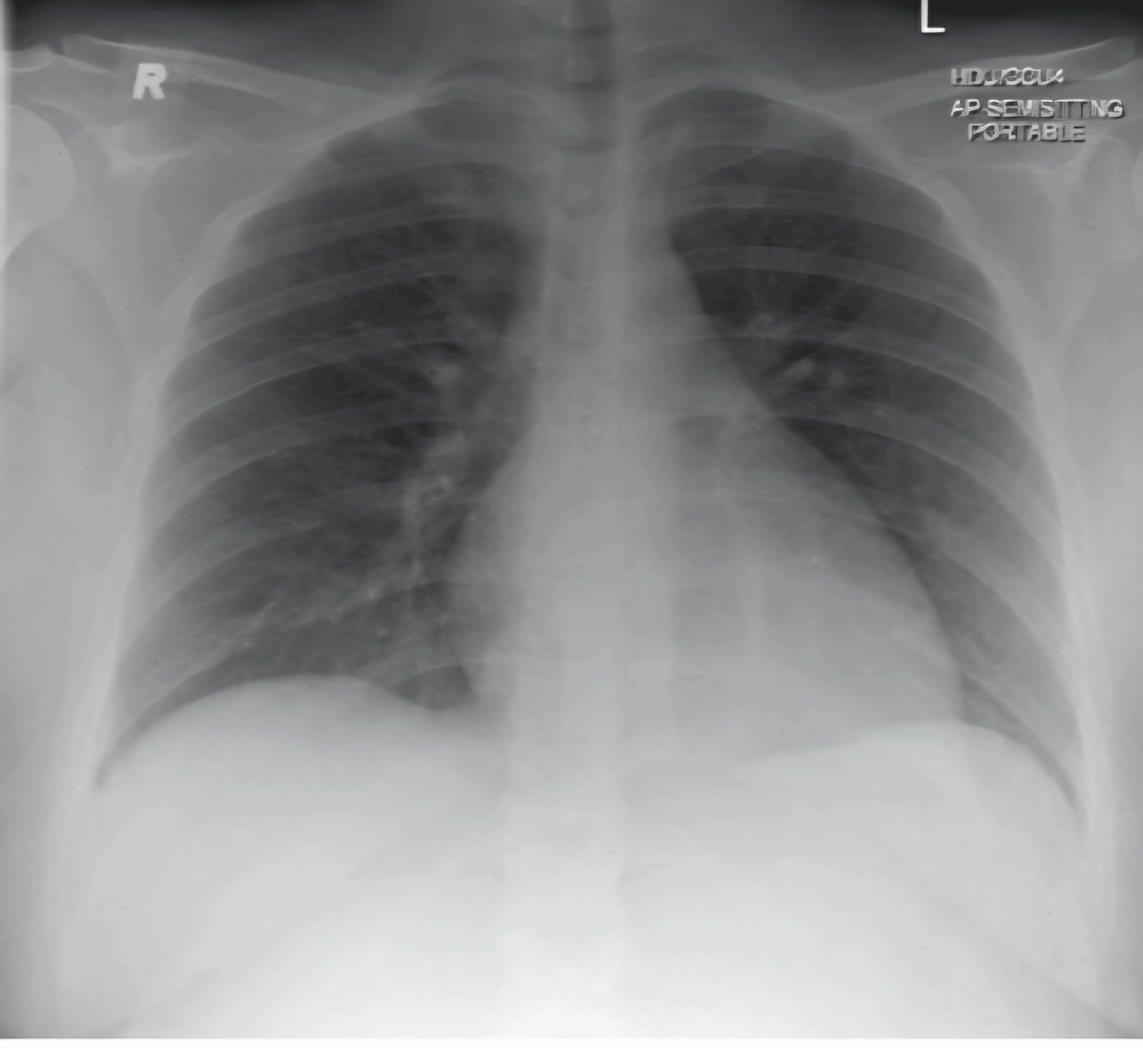
Repeat chest X-ray on day five showing resolution of the previously reported bronchopneumonia

On postoperative day five, she reported feeling well but still had a high-grade fever, and a repeat chest X-ray was performed, showing complete resolution of the previously reported bronchopneumonia (Figure 4). Despite the chest X-ray report, her antibiotics were changed to meropenem and teicoplanin after consulting with the infectious disease specialists, based on the assumption that her fever was likely due to pelvic infection, given her high risk for post-operative pelvic infection. 

**Figure 5:**
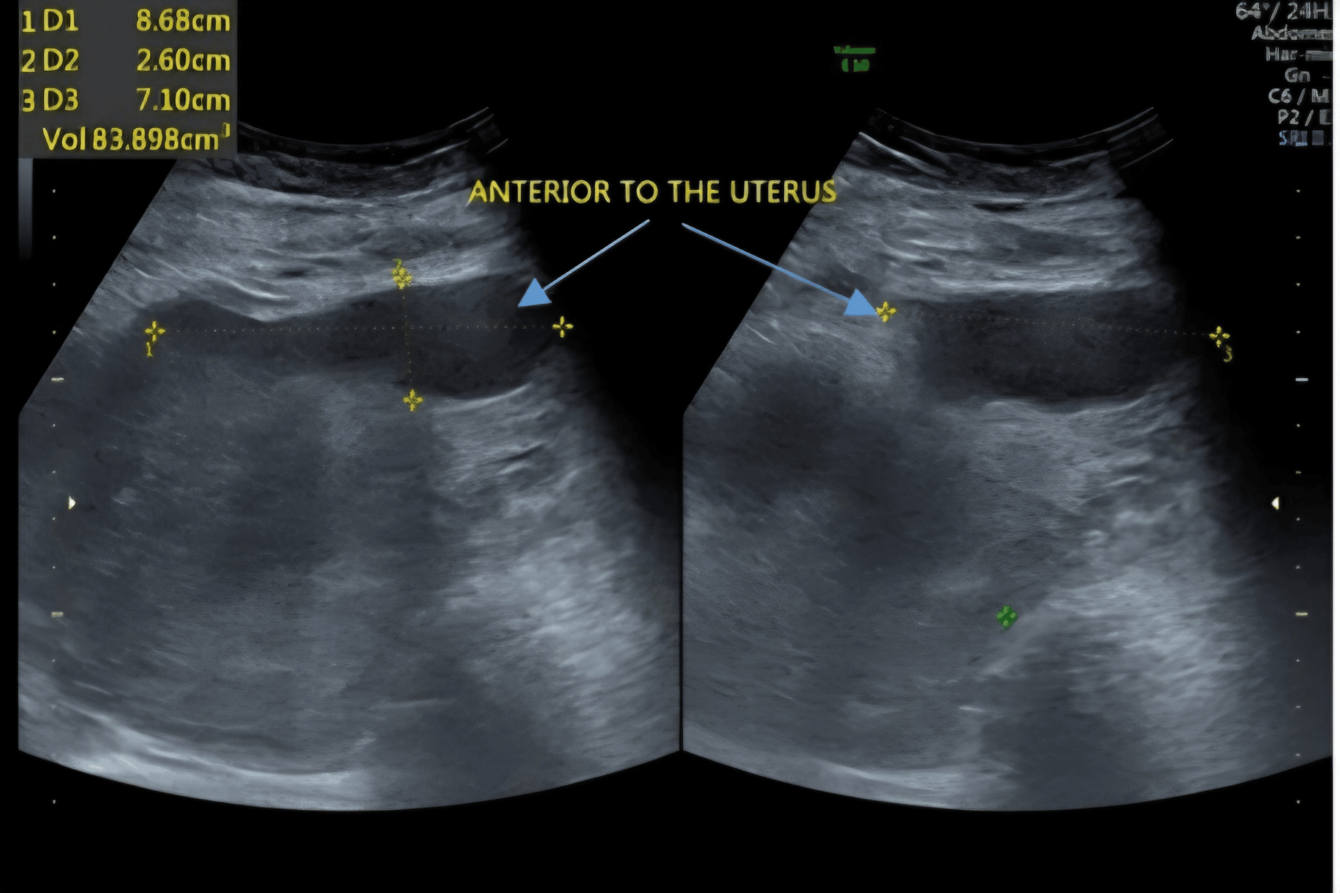
Hypoechoic collection anterior to the uterus

On postoperative day six, although her cough had improved significantly, and she reported feeling well, her high-grade fever persisted. A repeat abdominal and pelvic ultrasound was perfomed revealing increased amount of fluid in the abdominal cavity, a hypoechoic collection anterior to the uterus, measuring 86x71x26 mm (Figure 5) and a hypoechoic collection in the pouch of Douglas, measuring 23x32 mm. The possibility of urine extravasation due to bladder or ureteric injury was considered as a potential cause for the fluid collection but was excluded since she had no loin or abdominal pain, and her renal function tests and renal scan were normal. 

The persistent high-grade fever and worsening radiological findings raised the suspicion of worsening pelvic infection, leading to the patient's transfer to a tertiary hospital.

**Figure 6:**
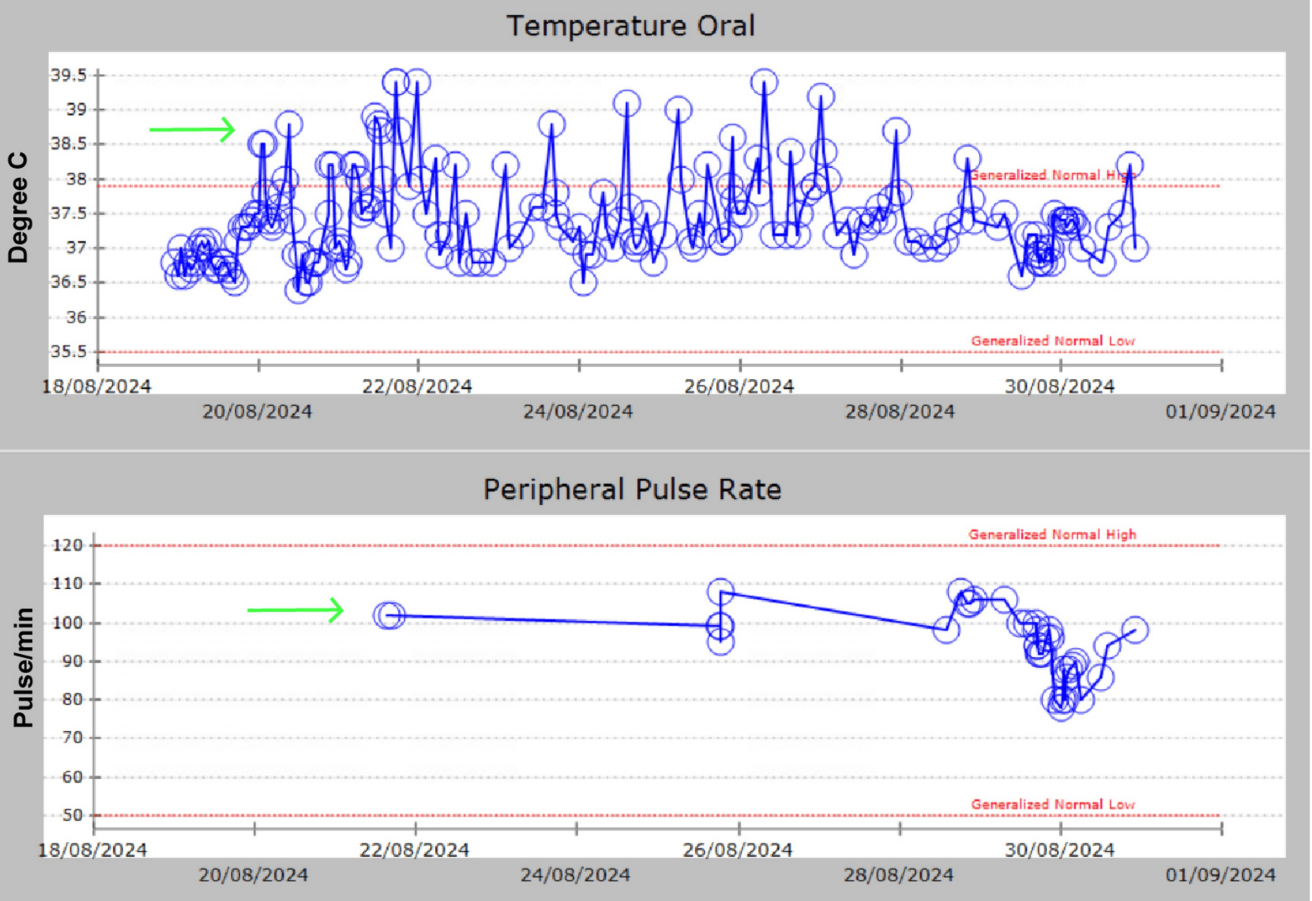
Temperature and peripheral pulse rate; the pulse rate remains around 100-110 beats per minute even when the temperature exceeds 39°C

At the tertiary hospital, she was cared for by an MDT of obstetricians, infectious disease specialists (ID specialists), physicians, radiologists, and pharmacists. A repeat renal, abdominal, and pelvic scan showed no new findings, nor did the repeat blood count. The fluid noted on the scan was not considered significant enough to require intervention as she did not report any abdominal pain or discomfort. Her temperature continues to fluctuate between 38°C and 39°C, and her pulse rate varies from the 80s to the 110s as shown in Figure 6. Therefore, her antibiotics were switched to piperacillin/tazobactam, although all her cultures returned negative and her WBC, lactic acid, and procalcitonin levels remained normal; the only elevated inflammatory marker was CRP, which is nonspecific for infection. The MDT believed that her persistent high-grade fever was likely caused by a pelvic infection related to her emergency caesarean section or infected abdominal fluid. They concluded that changing the antibiotic was a reasonable option in the absence of a positive culture, although she continued to be relatively unwell. 

**Figure 7:**
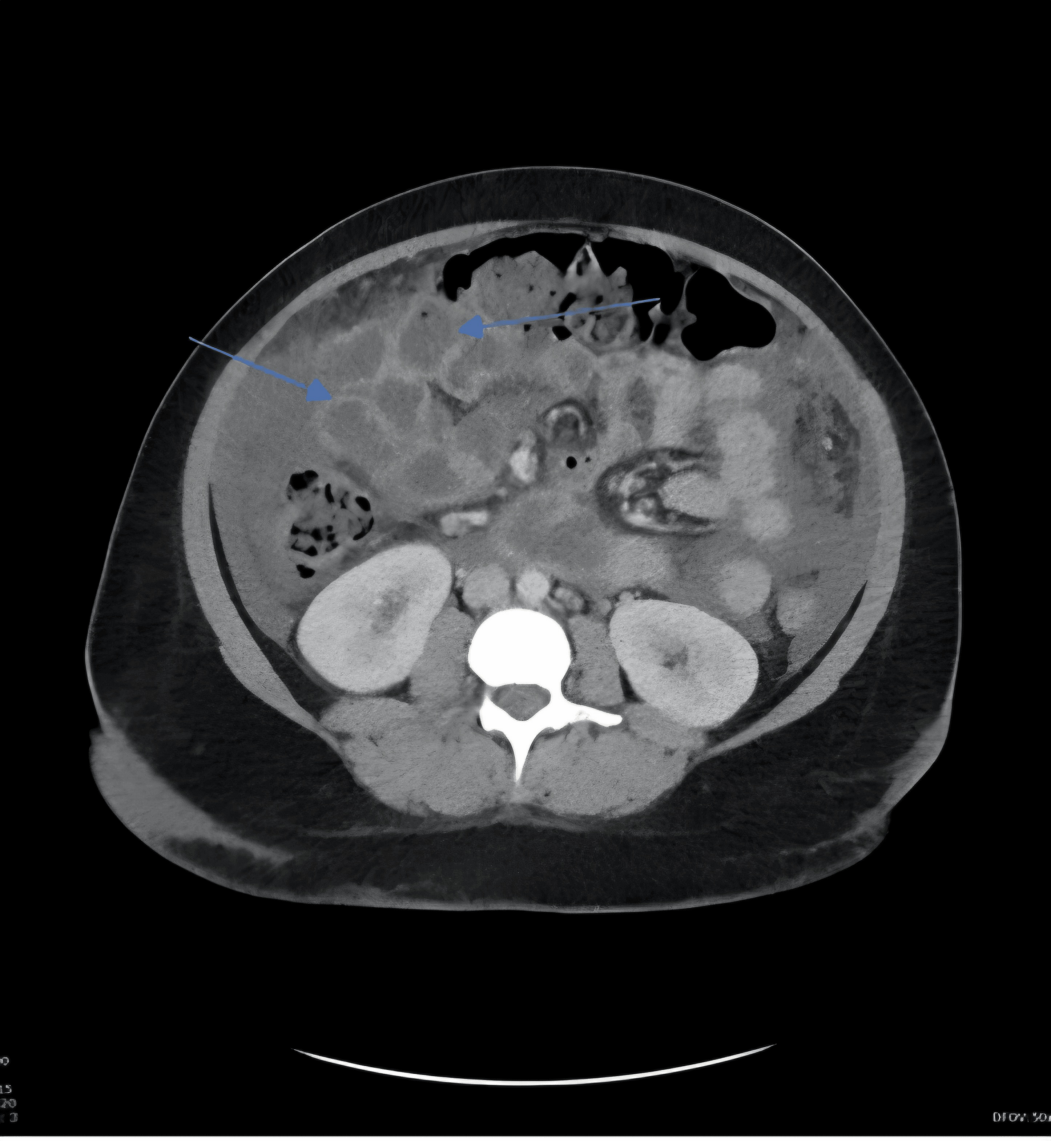
Computed tomography showing mesenteric oedema and peritoneal thickening

On postoperative day nine, she still had a high-grade fever of 39°C. A repeat CT of the abdomen and pelvis with contrast was performed to exclude pelvic vein thrombosis as the cause of the persistent fever. The scan showed an increase in free fluid within the abdominopelvic region, along with signs of mesenteric oedema, a collapsed small bowel with thickened wall and fat stranding along with peritoneal thickening, indicating worsening ongoing inflammatory changes (Figure 7).

However, there was no evidence of pelvic vein thrombosis. Doppler ultrasound of the lower limbs and an echocardiogram were performed and reported as normal. An MDT was convened, involving obstetricians, infectious disease specialists, and physicians, to discuss the CT scan report and develop a management plan in consultation with the patient and her family, taking into account her wishes. The team recognised that although the CT findings were highly suggestive of peritonitis, they felt it is unlikely to be peritonitis because aside from intermittent fever, the patient did not show any symptoms indicative of peritonitis. For instance, she consistently reported feeling well, had no abdominal pain, and was eating and drinking normally, with normal bowel movements. Repeated comprehensive assessments showed no signs such as abdominal tenderness, guarding, or rigidity that would indicate peritonitis. Her WBC, lactic acid, and procalcitonin were consistently normal, with CRP the only raised inflammatory marker. After extensive discussion, the team's decision was to proceed with an exploratory laparotomy, as she had already tried different antibiotics. The plan was discussed with the patient and her family, but her family declined surgery, stating that she is well apart from the fever and wished to continue with antibiotics. The MDT team accepted their decision.

**Figure 8:**
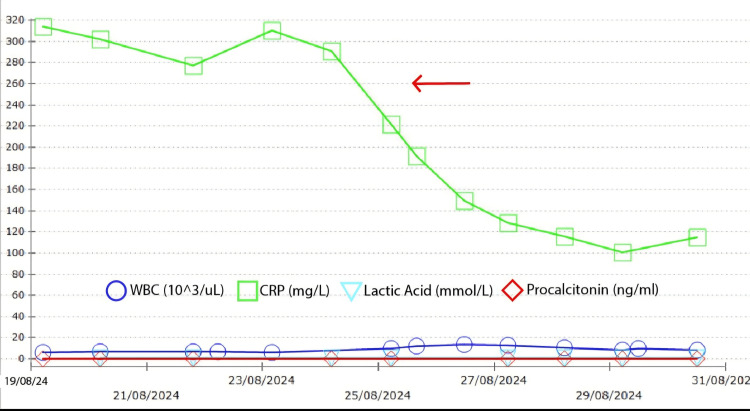
WBC, lactic acid, and procalcitonin levels were mostly within normal limits, whereas CRP was elevated and returned to normal once antibiotics were stopped WBC: white blood cell count, CRP: C-reactive protein

Her white cell count, lactic acid, and procalcitonin remained within normal limits throughout, apart from CRP, which was the only elevated inflammatory marker but quickly returned to normal once her antibiotics were discontinued, as shown in Figure 8.

However, the following day after the CT report (postoperative day 10), she was observed to be jaundiced. Her haemoglobin level was 8.0 g/L, WBC count 12.4x10^3/uL, lactic acid 1.1 mg/L, alanine aminotransferase (ALT) 234 U/L (reference range <32 U/L), aspartate aminotransferase (AST) 237 U/L (reference range <32 U/L), and bilirubin 59 μmol/L (reference range <21 μmol/L), as shown in Table 1.

A diagnosis of drug-induced hepatitis and possibly drug-induced fever was made. Her antibiotics were discontinued, and she was prescribed only paracetamol. Within 48 hours of stopping all antibiotics, her temperature became less frequent and started to normalise. A decision was made to transfuse two units of packed RBC transfusion to aid her recovery. She was discharged on day 16 after remaining afebrile for more than 24 hours, and all her blood tests were trending towards normal. She was reviewed in the clinic four weeks later and reported feeling well with no further episodes of fever, and all her blood investigations had returned to normal. 

## Discussion

Drug-induced fever is an important yet often overlooked differential diagnosis for persistent fever in obstetric care. It can be difficult to identify when patients are on antibiotics for suspected postoperative infections. The global rate of caesarean sections has continued to rise, from about 7% in 1990 to 21% today [8]. Although this procedure can be lifesaving, it is associated with increased maternal complications, including haemorrhage, thrombosis, and puerperal infections [9], with emergency caesarean sections posing a higher risk of complications than elective ones. Caesarean delivery remains the leading cause of postpartum infection, with the global incidence of SSI following caesarean at 7.0%, and 9-11% in the UK [2,3]. Persistent fever in a postoperative patient is a major concern, as it may indicate an ongoing or worsening infection that requires systematic evaluation and a multidisciplinary approach.

Our patient underwent a comprehensive evaluation, including urine and blood cultures, a viral screen, pelvic ultrasound, CT pelvis and chest X-ray, for an infectious cause for her fever. Broad-spectrum antibiotics were started after chest imaging indicated bronchopneumonia, and pelvic scan and CT showed a small pelvic hematoma. Persistent fever requires multidisciplinary management and additional imaging to exclude pelvic or surgical site infection, infected pelvic vein thrombosis, or pulmonary embolism.

Drug-induced fever is often overlooked in the initial diagnosis of persistent fever in postoperative patients because the clinical features vary considerably, and infection is also a major concern after any operation, especially unplanned emergency procedures such as emergency caesarean section, as in our case. Antibiotics are among the most common medications responsible for drug fever, with β-lactam antibiotics frequently implicated. The pathophysiology involves immune-mediated hypersensitivity reactions that release inflammatory cytokines, including interleukin-1 and interleukin-6, which stimulate the hypothalamic thermoregulatory centre. Consequently, laboratory abnormalities such as elevated CRP may be present, but the absence of infection is reflected by normal WBC, lactic acid, and procalcitonin levels. 

Therefore, a high degree of suspicion is necessary to diagnose drug-induced fever early in patients with ongoing fever. Some clinicians have reported a discrepancy between the patient's overall condition and the high-grade fever, with patients reporting an unusual sense of well-being despite the high temperature [10]. This was observed in our patient, and in hindsight, we should have considered drug-induced fever as early as day five, when, despite a high-grade fever, the patient felt well, examination showed no significant abnormalities, and a repeat chest X-ray confirmed the resolution of the previously diagnosed bronchopneumonia.

Some studies have reported a high incidence of pulse deficit or pulse dissociation in patients with drug-induced fever, non-infectious diseases, or those using beta-blockers [11]. According to Liebermeister, for each degree Celsius above 38.3°C (101°F), the heart rate increases by approximately 10 beats per minute [12]. However, this rise in heart rate in response to fever can often be absent in drug fever. Faget’s sign [13] describes an inverse relationship between body temperature exceeding 38.3°C (101°F) and pulse rate. Mackowiak et al. [14] reported that over 83% of patients with drug fever exhibit relative bradycardia. Our patient's pulse rate was 110 or less for most of the time, even when her temperature was above 39.5°C, as shown in Figure 6. However the pulse rate was not documented every four hours as planned, leading to insufficient data to draw a definitive conclusion about relative bradycardia.

Radiological findings in drug-related inflammatory reactions are often nonspecific and can mimic intra-abdominal infection, which can lead to a diagnostic dilemma and prolongation of therapy if drug fever is not considered. Our patient's CT abdomen and pelvis one day before she developed drug-induced hepatitis, which led to us stopping all her antibiotics, showed a potentially worsening pelvic infection and possible peritonitis (Figure 7). The CT findings did not align with the patient's ongoing report of feeling well apart from intermittent fever and did not show any symptoms indicative of peritonitis. For instance, she had no abdominal pain, and was eating and drinking normally, with normal bowel movements. Repeated comprehensive assessments showed no signs such as abdominal tenderness, guarding, or rigidity that would indicate peritonitis. The discrepancy between the imaging report and her sense of well-being, combined with her normal WBC, procalcitonin, and lactic acid levels, created a diagnostic and management dilemma. Although we felt the patient was generally well apart from high-grade intermittent fever, we could not ignore the CT report. The MDT plan was for an exploratory laparotomy, which is not without risks. A detailed discussion was held with the patient and her family, explaining the benefits and risks of laparotomy. This is essential to ensure a shared decision, especially when the diagnosis remains uncertain. 

The ultrasound and CT findings highlight the limitations and pitfalls of imaging in patient management and emphasise the need for awareness and vigilance regarding discrepancies between imaging results and the patient's clinical condition. We might have caused harm by performing unnecessary laparotomy had we relied solely on the CT report and not considered our patient's overall condition or involved the patient and her family in discussions of her care plan, which led to her decision to continue with medical treatment. About one in 10 patients experiences harm in healthcare, with over half of these cases being preventable, and half involving medication [15,16]. The development of jaundice was a significant turning point because it led to the diagnosis of drug-induced hepatitis, and for the first time, the possibility of antibiotic-induced fever was considered.

Our patient was receiving ceftriaxone, which was later switched to piperacillin/tazobactam, both of which are β-lactam antibiotics that are commonly associated with drug fever. β-lactam antibiotics share a β-lactam ring structure, which can act as a hapten and bind to proteins in the body to trigger immune complex hypersensitivity reactions and fever. Because the β-lactam class of antibiotics share the same structural core, any β-lactam antibiotic can cause drug fever, and this effect is not limited to a single agent. The timing of onset and the fever pattern can vary widely, from a few hours to days or even months after starting the drug, further complicating the diagnosis of drug-induced fever [14,17]. After discontinuing the offending drug, complete resolution of the fever may take several days. In our case, her fever became less frequent the day after we stopped all her antibiotics, and she became apyrexial within 48 hours. However, in some cases, fever can persist for days or weeks if the reaction is accompanied by other hypersensitivity symptoms or if the drug is eliminated slowly.

Finding normal WBC, procalcitonin, and lactic acid levels is valuable in differentiating drug fever from infection-related fever. Procalcitonin and lactic acid are key biomarkers for diagnosing bacterial infections and distinguishing bacterial causes of fever from non-infectious ones. An elevated CRP, as seen in our case (Table 1), alongside normal WBC, procalcitonin, and lactic acid, is more consistent with a systemic cytokine-mediated inflammatory response than with infection. These findings, combined with her feeling generally well despite a high-grade fever, should have alerted us on postoperative day five to the possibility of drug-induced fever as a differential diagnosis. The MDT also assessed the clinical significance of the ultrasound finding of increased intra-abdominal fluid as a possible cause of her fever, but she has no abdominal symptoms, and the abdominal examination was normal, with no signs of distension, tenderness, or fluid shift. Given her final diagnosis of antibiotic-induced fever, the increased abdominal fluid could possibly be her body's systemic response to hypersensitivity immune complex reaction caused by the drug-fever. 

Diagnosing antibiotic-induced fever in our case was quite challenging because we started antibiotics due to a suspected infection, making it difficult to distinguish between fever caused by antibiotics and an ongoing postoperative infection. Moreover, her repeat pelvic ultrasound showed an increase in intra-abdominal fluid and abdominal and pelvic CT scan was highly suggestive of worsening pelvic infection, including peritonitis (Figure 7). However, our patient reported feeling well apart from intermittent fever and had no symptoms suggestive of peritonitis. Repeated comprehensive clinical assessments also did not show abnormalities to suggest peritonitis. Her antibiotics were stopped once a diagnosis of drug-induced hepatitis was made. We expected her condition to worsen after stopping antibiotics and without surgical intervention if peritonitis had been the cause of her fever and the CT findings. However, her fever became less frequent and eventually ceased. We firmly believe that CT changes were due to unusual systemic hypersensitivity immune complex reactions - namely, a drug fever caused by β-lactam antibiotic administration. 

Causality was further assessed using the WHO-UMC Causality Assessment System [18], which classified the adverse reaction as probable/likely, based on the temporal relationship with drug exposure, the absence of alternative explanations, and rapid defervescence after drug withdrawal. Similarly, application of the Naranjo Adverse Drug Reaction Probability Scale [19] resulted in a score of 7, indicating a probable adverse drug reaction. Rechallenge with the suspected drug was not carried out due to ethical considerations.

## Conclusions

Postoperative fever is not unusual in obstetric care, and identifying its cause can be challenging. Nosocomial infections, pelvic infections, and SSIs are among the main sources of postoperative infections. Antibiotic-induced fever is rare in obstetrics and is often overlooked in the initial differential diagnosis of persistent postoperative fever. It can also be difficult to recognise when patients are receiving antibiotics for suspected postoperative infections. β-lactams are frequently used for postpartum infections and are most often linked to drug fever. As the global caesarean rate increases, obstetricians are more likely to encounter cases where antibiotic-induced fever overlaps with infection-related fever during the puerperium.

A systematic, multidisciplinary approach is vital for early diagnosis and for achieving good clinical outcomes in patients with persistent fever after delivery due to a systemic inflammatory process driven by hypersensitivity immune complex reactions. Abdominal and pelvic imaging results are often nonspecific and can resemble infectious conditions, leading to diagnostic dilemmas. Awareness, vigilance, and clinical correlation are essential when diagnosing drug-induced fever. Delayed diagnosis can result in longer hospital stays, unnecessary tests, increased healthcare costs, and harm to the patient.
